# Shape-Induced Terminal Differentiation of Human Epidermal Stem Cells Requires p38 and Is Regulated by Histone Acetylation

**DOI:** 10.1371/journal.pone.0027259

**Published:** 2011-11-02

**Authors:** John T. Connelly, Ajay Mishra, Julien E. Gautrot, Fiona M. Watt

**Affiliations:** 1 Barts and the London School of Medicine and Dentistry, Queen Mary University of London, London, United Kingdom; 2 Cambridge Research Institute, Cancer Research UK, Cambridge, United Kingdom; 3 School of Engineering and Materials Science, Queen Mary University of London, London, United Kingdom; 4 Wellcome Trust Centre for Stem Cell Research, University of Cambridge, Cambridge, United Kingdom; Dalhousie University, Canada

## Abstract

Engineered model substrates are powerful tools for examining interactions between stem cells and their microenvironment. Using this approach, we have previously shown that restricted cell adhesion promotes terminal differentiation of human epidermal stem cells via activation of serum response factor (SRF) and transcription of AP-1 genes. Here we investigate the roles of p38 MAPK and histone acetylation. Inhibition of p38 activity impaired SRF transcriptional activity and shape-induced terminal differentiation of human keratinocytes. In addition, inhibiting p38 reduced histone H3 acetylation at the promoters of SRF target genes, *FOS* and *JUNB*. Although histone acetylation correlated with SRF transcriptional activity and target gene expression, treatment with the histone de-acetylase inhibitor, trichostatin A (TSA) blocked terminal differentiation on micro-patterned substrates and in suspension. TSA treatment simultaneously maintained expression of *LRIG1*, *TP63*, and *ITGB1*. Therefore, global histone de-acetylation represses stem cell maintenance genes independent of SRF. Our studies establish a novel role for extrinsic physical cues in the regulation of chromatin remodeling, transcription, and differentiation of human epidermal stem cells.

## Introduction

Cell-extracellular matrix (ECM) interactions are key regulators of epidermal stem cell fate. Epidermal stem cells express high levels of β1 [1], [2] and α6 [3] integrins, and integrin-mediated adhesion to the basement membrane maintains keratinocytes in an undifferentiated state [4]. In vitro, loss of adhesion to the ECM rapidly induces epidermal terminal differentiation [5]. We recently developed novel micro-patterned substrates on which ECM proteins are deposited in defined shapes and areas [6], [7]. Using this system to precisely control the level of adhesion and spreading of single cells, we demonstrated that limited cell adhesion promotes cortical actin polymerization and terminal differentiation in human epidermal stem cells. Downstream of actin polymerization, differentiation is regulated by serum response factor (SRF) and AP-1 transcription factors. The SRF signaling pathway therefore links extrinsic physical cues to a transcriptional mechanism for controlling cell fate within the epidermis [6], [8], [9].

SRF is a transcription factor expressed in many tissues, and its activity is modulated by interactions with various co-factors, including myocardin-related transcription factor-A (MRTF-A; also known as MAL, MKL1) [10]. Monomeric (G-) actin binds MRTF-A and inhibits nuclear accumulation and activation of SRF. F-actin polymerization releases MRTF-A to enter the nucleus and co-activate transcription with SRF [10], [11]. SRF targets include several adhesion-related genes (*ACTB*, *VCL*) and immediate early genes (*FOS*, *JUNB*, *EGR1*) [11], [12]. In addition to actin polymerization, SRF can be activated by the mitogen activated protein kinase (MAPK), extracellular signal-related kinase (ERK) [13]. This pathway stimulates a distinct set of co-factors, which for some genes antagonize MRTF-A interactions with SRF [14], [15]. Thus, specific SRF target genes exhibit differential sensitivity to cytoskeletal organization and MRTF-A activation.

Several recent reports demonstrate that epigenetic changes in chromatin remodeling can have a profound effect on epidermal development and homeostasis. Polycomb repressive complexes (PRCs) silence both senescence and terminal differentiation genes by tri-methylating lysine 27 of histone H3 (triMeK27-H3) [16]. This specific mark aids chromatin packing and limits transcription [17]. In contrast, histone tail acetylation promotes an open chromatin structure and allows transcription factors to more easily access the DNA [17]. Histone deacetylases (HDAC) 1 and 2 are required for normal terminal differentiation and repression of p16/Ink4a in the developing mouse epidermis [18]. In adult epidermis, histone acetylation also promotes exit from the hair follicle stem cell compartment, and epidermal stem cells display hypo-acetylated H3 and H4 [18], [19]. While it is clear that chromatin remodeling plays an important role in epidermal homeostasis, little is known about the upstream or extrinsic signals that control the activation and specificity of chromatin remodeling complexes.

In the present study, we sought to determine whether the physical regulation of human keratinocyte differentiation depends on intrinsic signaling pathways in addition to actin polymerization. Since SRF activity is sensitive to both cytoskeletal organization and MAPK signaling, we examined how various MAP kinases influence terminal differentiation using micro-patterned substrates and whether chromatin modification is involved.

## Results

### p38 signaling is required for shape-induced differentiation

**Figure 1:**
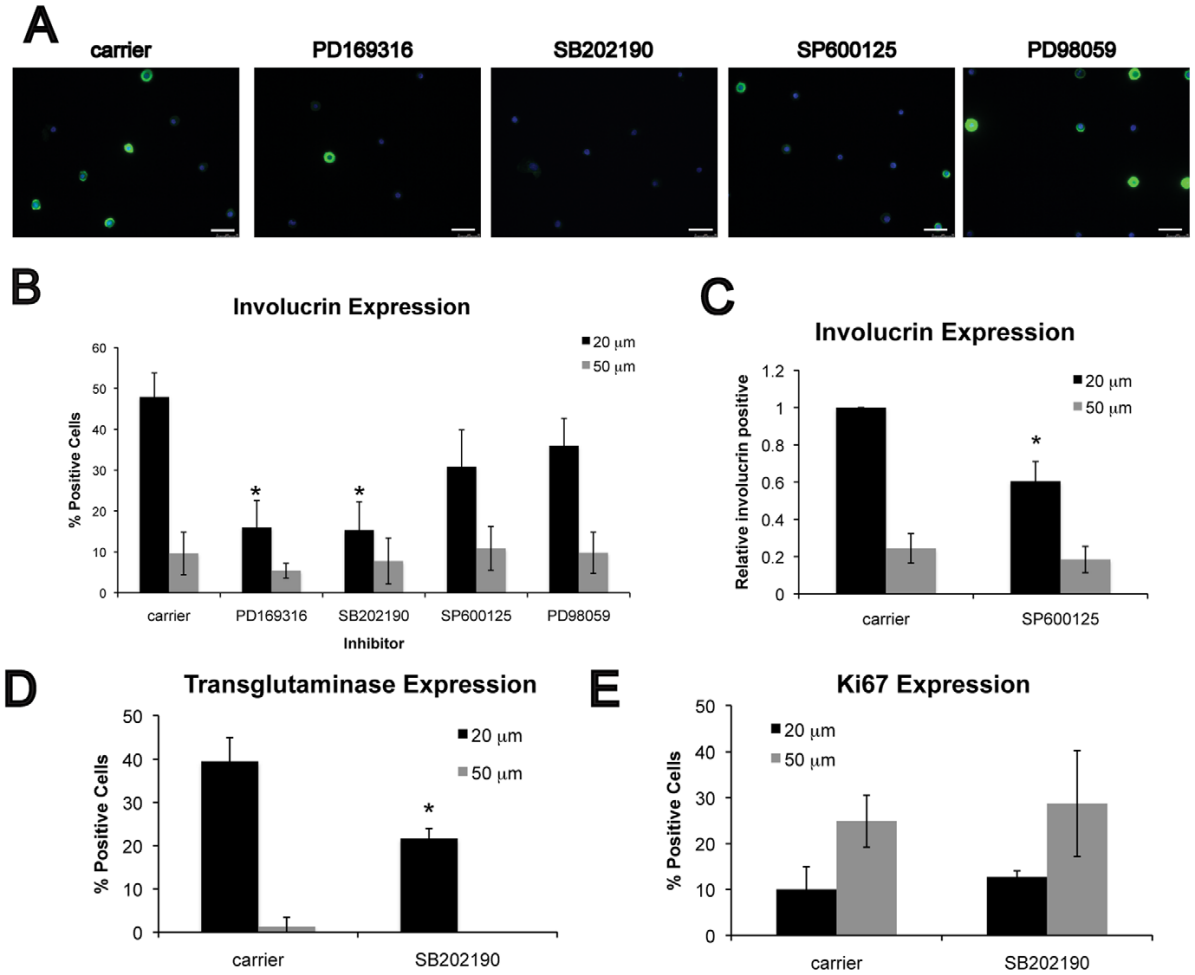
Effect of MAPK inhibitors on shape-induced terminal differentiation. (A) Representative images of keratinocytes on micro-patterned substrates comprising 20 µm diameter collagen islands. Cells were cultured for 24 h in medium containing carrier (0.1% DMSO), 10 µM PD169316, 2 µM SB202190, 10 µM SP600125, or 10 µM PD98059. Immunofluorescence staining for involucrin (green) shows terminally differentiating cells, and nuclei are labeled with DAPI (blue). Scale bar  = 50 µm. Quantification of (B-C) involucrin, (D) transglutaminase I, and (E) Ki67 positive cells after 24 h on 20 µm or 50 µm substrates. In (C), the proportion of involucrin positive cells was normalized to carrier-treated cells on 20 µm islands. Data are means ± SEM of 3 experiments. *P<0.05 compared to carrier.

To investigate the role of MAP kinase signaling in shape-induced terminal differentiation, primary human keratinocytes were seeded onto 20 µm or 50 µm diameter collagen-coated circular islands. Cells were cultured on the substrates for 24 h in the presence or absence of p38, JNK, and ERK inhibitors. Consistent with our previous findings [6], cells on 20 µm islands were unable to spread and approximately 50% initiated expression of the terminal differentiation marker, involucrin, whereas fewer than 10% of cells on 50 µm islands expressed involucrin (Fig. 1A, B).

Treatment with both p38 inhibitors, PD169316 and SB202190, significantly reduced the number of involucrin positive cells, but inhibiting ERK activity with PD98059 did not affect involucrin expression (Fig. 1A, B). Similarly, we observed no effect of the MEK inhibitor, U0126, on shape-induced differentiation [6]. The decrease in the absolute number of involucrin-positive cells in the presence of the JNK inhibitor, SP600125, was not statistically significant (Fig. 1A–B); however, when normalized to involucrin expression in carrier-treated cells there was a small but significant reduction (Fig. 1C). Inhibition of p38 MAPK not only inhibited involucrin expression, but also inhibited shape-induced expression of transglutaminase I (Fig. 1D). We previously showed that cell shape-induced differentiation is independent of inhibition of proliferation [6], [20]; consistent with those results, p38 inhibition did not affect the proportion of Ki67 positive cells (Fig. 1E). We conclude that p38 activity is required for shape-induced differentiation in human keratinocytes.

### p38 mediates SRF transcriptional activity

**Figure 2:**
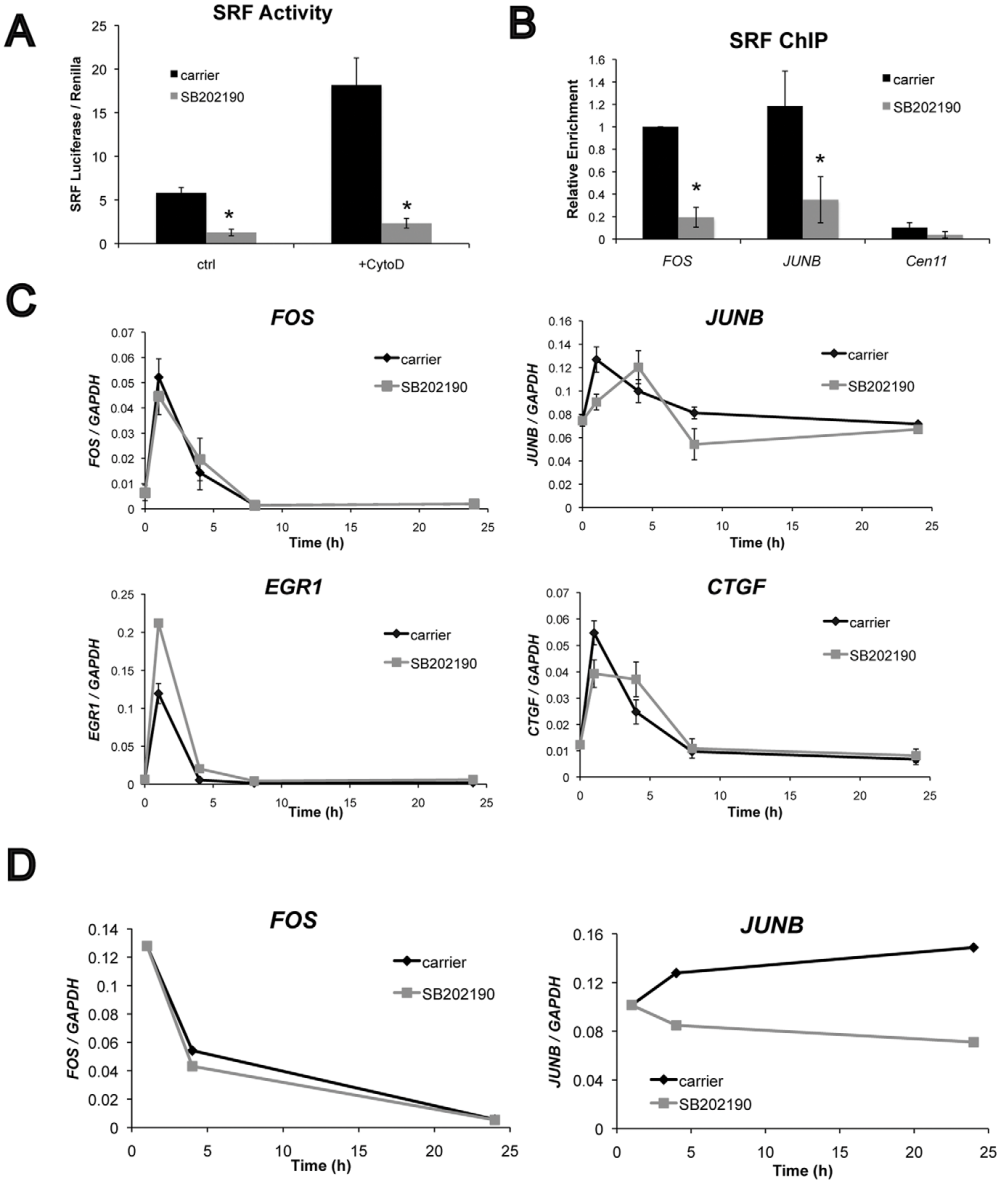
Effect of p38 inhibition on SRF signaling. (A) SRF transcriptional activity in human keratinocytes expressing the SRF luciferase reporter and TK renilla control. Cells were treated overnight with 2 µM SB202190 or carrier, then 1 µM cytochalasin D or carrier for 7 h. Data are means ± SEM of 4 replicates from one experiment (representative of 3 independent experiments). *P<0.05 compared to carrier. (B) ChIP for SRF following 24 h treatment with 2 µM SB202190. SRE regulatory elements in the *FOS* and *JUNB* genes were detected by real-time RT-PCR, and the centromeric region of chromosome 11 (*SATCEN11*) was used as a negative control. Enrichment of the anti-SRF IP is compared to anti-FLAG control, then normalized to *FOS* carrier condition. Data are means ± SEM of 3 experiments. *P<0.05 compared to carrier. (C) Expression of endogenous SRF target genes measured by real-time RT-PCR. Cells were serum-starved overnight with or without 2 µM SB202190, then stimulated with 10% FBS-containing medium for 24 h ±2 µM SB202190. Data are means ± SEM of 3 experiments. (D) Expression of endogenous *FOS* and *JUNB* on micro-patterned substrates. Cells were seeded onto 20 µm islands and allowed to adhere for 1 h before rinsing and treating with carrier or 2 µM SB202190 for an addition 23 h.

Since SRF promotes keratinocyte terminal differentiation in response to restricted adhesion, we investigated whether p38 inhibition influenced SRF signaling. SB202190 treatment of keratinocytes on non-patterned surfaces significantly inhibited SRF reporter activity in dual luciferase assays (Fig. 2A) and reduced the amount of SRF bound to the promoters of the SRF target genes, *FOS* and *JUNB* (Fig. 2B). To understand how p38 regulates the expression of endogenous SRF target genes, keratinocytes were serum starved overnight in the presence or absence of SB202190, then stimulated with 10% FBS-containing medium for 24 h. Under control conditions, each of the SRF target genes, *FOS, JUNB, EGR1,* and *CTGF*, peaked at 1 h following stimulation (Fig. 2C). Despite reducing SRF binding to the *FOS* promoter, treatment with SB202190 had no effect on *FOS* expression (Fig. 2C). SB202190 delayed the activation of *JUNB* and *CTGF* (Fig. 2C). While the kinetics of *EGR1* activation were unaffected by p38 inhibition, the peak level increased significantly (Fig. 2C).

**Figure 3:**
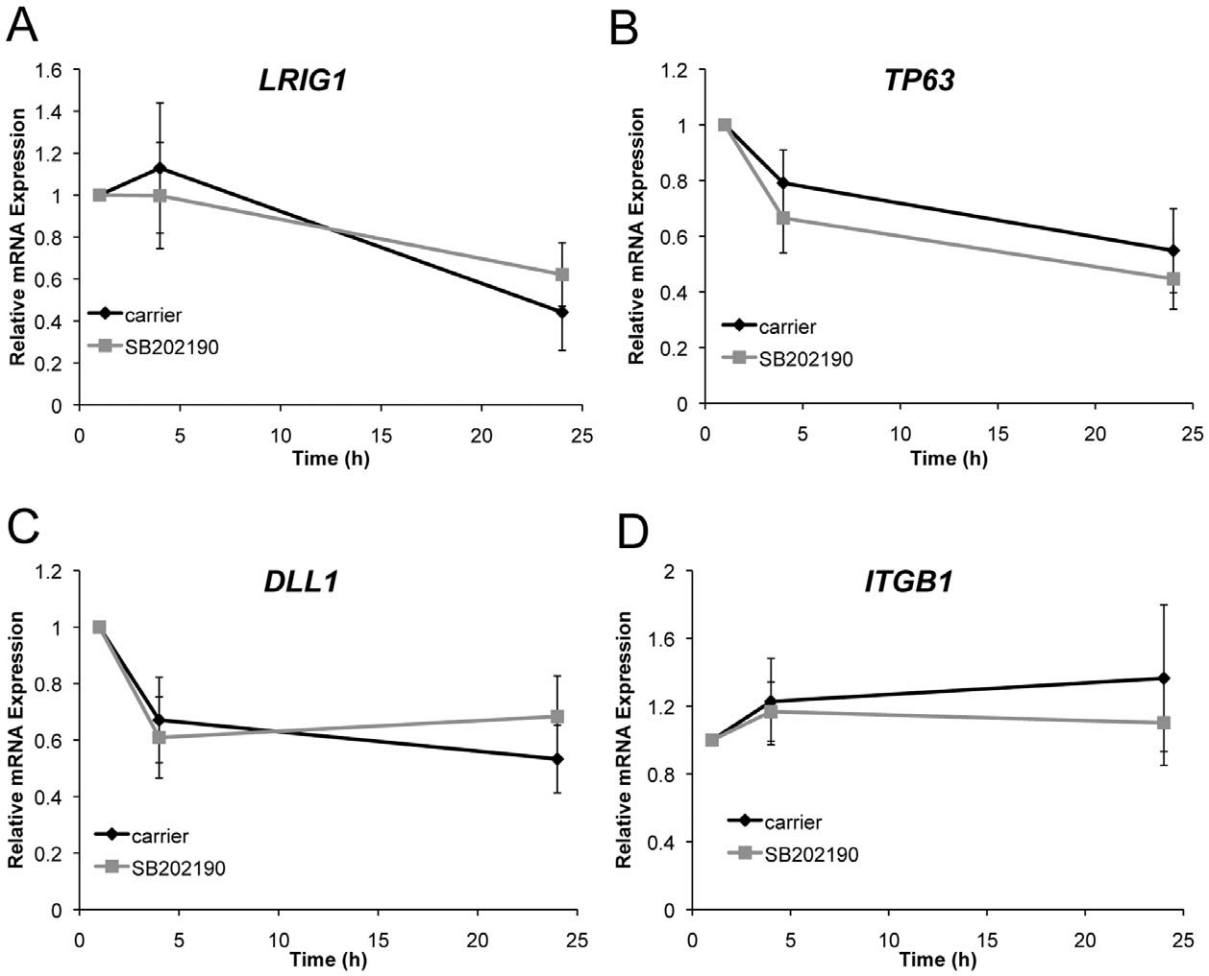
Effect of p38 inhibition on epidermal stem cell gene expression. Real-time RT-PCR detection of (A) *LRIG1*, (B) *TP63*, (C) *DLL1*, and (D) *ITGB1* mRNA levels for cells on micro-patterned substrates. Cells were seeded onto 20 µm islands and allowed to adhere for 1 h before rinsing and treating with carrier or 2 µM SB202190. Expression levels were normalized to the 1 h time point and represent the means ± SEM of 3 experiments.

We also examined expression of AP-1 and stem cell regulatory genes during shape induced differentiation on the micro-patterned substrates. When keratinocytes were cultured on 20 µm islands, SB202190 treatment had little effect on *FOS* expression; however, p38 inhibition reduced *JUNB* activation by approximately 50% at 4 and 24 hours after seeding (Fig. 2D). The stem cell markers *LRIG1* [21], *DLL1* [22], and *TP63* [23] were all down regulated 24 hours after seeding onto 20 µm islands, and inhibition of p38 had little effect on expression of these genes (Fig. 3A–C). Given that *ITGB1* is a direct SRF target [24], it is interesting to note that *ITGB1* levels were maintained over 24 hours, with or without SB202190 (Fig. 3D). Taken together, these results indicate that during shape-induced differentiation, p38 specifically regulates *JUNB*, but not the stem cell markers *LRIG1*, *DLL1*, and *TP63*.

**Figure 4:**
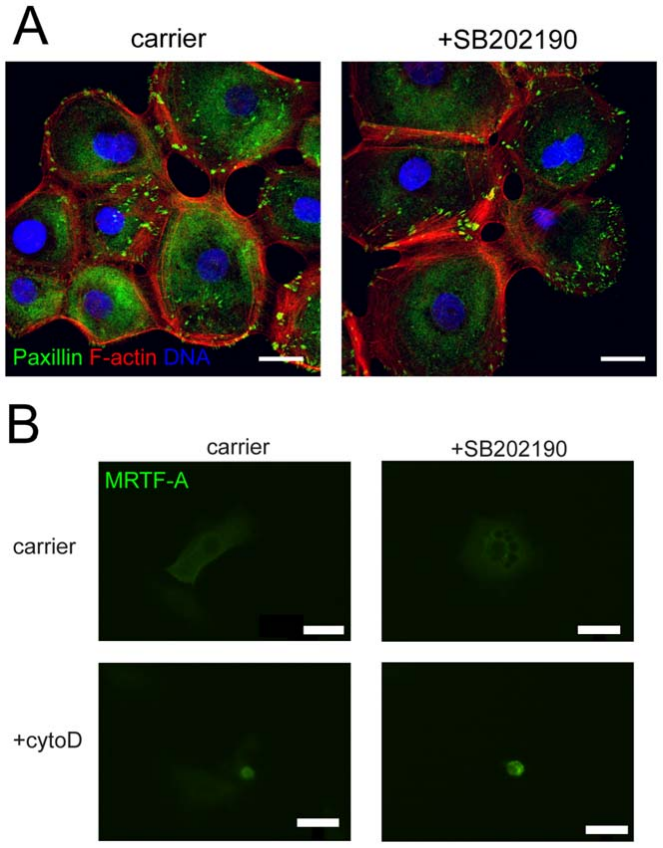
Effect of p38 inhibition on cytoskeletal organization and MRTF-A localization. (A) Representative images of paxillin (green) and F-actin (red) localization in keratinocytes treated with carrier or 2 µM SB202190 for 24 h. Scale bar  = 20 µm (B) Representative image of MRTF-A localization in keratinocytes transfected with FLAG-MRTF-A construct, treated overnight with or without SB202190 and then stimulated with 1 µM cytochalasin D for 1 h. MRTF-A was detected by immunofluorescence staining for the FLAG tag. N = 20 cells examined per condition. Scale bar  = 50 µm.

The SRF target genes that were affected by SB202190, *JUNB* and *CTGF*, are both regulated by the actin cytoskeleton and MRTF-A [25]. We therefore investigated whether p38 signaling directly affected cytoskeletal organization and MRTF-A localization. On non-patterned surfaces, inhibition of p38 activity with SB202190 did not alter the formation of paxillin containing focal adhesions or the assembly of F-actin fibers (Fig. 4A). Furthermore, MRTF-A nuclear translocation in response to cytochalsin D treatment [6] was not affected by p38 inhibition (Fig. 4B). These results suggest that p38 influences transcription via altered interactions between SRF and MRTF-A inside the nucleus.

### Histone acetylation influences SRF transcriptional activity and is regulated by p38

**Figure 5:**
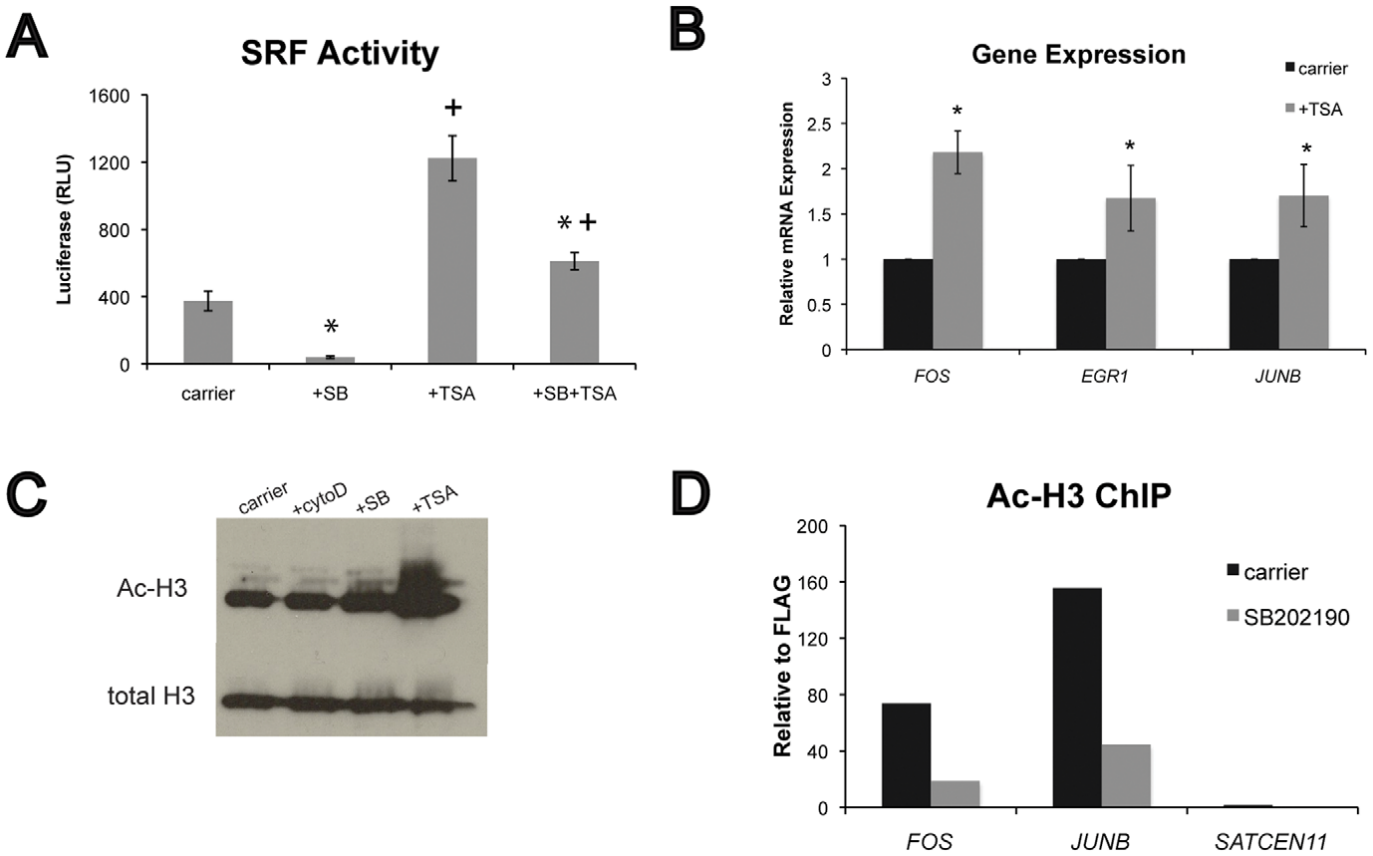
Role of histone acetylation in SRF signaling. (A) SRF transcriptional activity in human keratinocytes expressing the SRF luciferase reporter and TK renilla control. Cells were treated overnight with or without 2 µM SB202190 or 200 nM TSA, then stimulated for 7 h with 10% FBS. Data are means ± SEM of 4 replicates from one experiment (representative of 3 independent experiments). *P<0.05 for effect of SB202190, +P<0.05 for effect of TSA. (B) Expression of endogenous SRF target genes measured by real-time RT-PCR. Cells were serum-starved overnight with or without 200 nM TSA, then stimulated with 10% FBS-containing medium for 1 h. Data are means ± SEM of 3 experiments. *P<0.05 compared to carrier. (C) Western blot of total and acetylated histone H3 in cells after 24 h treatment with carrier, 1 µM cytochalasin D, 2 µM SB202190, or 200 nM TSA. (D) ChIP of acetylated H3 in cells following 24 h treatment with 2 µM SB202190. DNA sequence flanking the SRE regulatory elements in the *FOS* and *JUNB* genes were detected by real-time RT-PCR, and the centromeric region of chromosome 11 (*SATCEN11*) was used as a negative control. Enrichment from the anti-AcH3 IP is reported relative to anti-FLAG control.

Global changes in histone acetylation influence epidermal stem cell self-renewal and terminal differentiation [18], and p38 has recently been linked to SRF signaling and histone acetylation [26]. We therefore examined the effect of histone acetylation on SRF transcriptional activity in human keratinocytes. Treatment for 24 h with the histone de-acetylase inhibitor, trichostatin A (TSA) significantly enhanced SRF transcriptional activity in luciferase assays (Fig. 5A). Similarly, we observed a 1.5–2 fold increase in *FOS*, *EGR1*, and *JUNB* expression following TSA exposure (Fig. 5B). Cytoskeletal disruption with cytochalasin D or p38 inhibition with SB202190 had little effect on global histone acetylation (Fig. 5C). However, SB202190 treatment specifically reduced histone acetylation at the *FOS* and *JUNB* promoters (Fig. 5D). Based on these results, we conclude that histone acetylation promotes SRF transcriptional activity and is mediated by p38 signaling.

### Global histone de-acetylation is required for shape-induced differentiation

**Figure 6:**
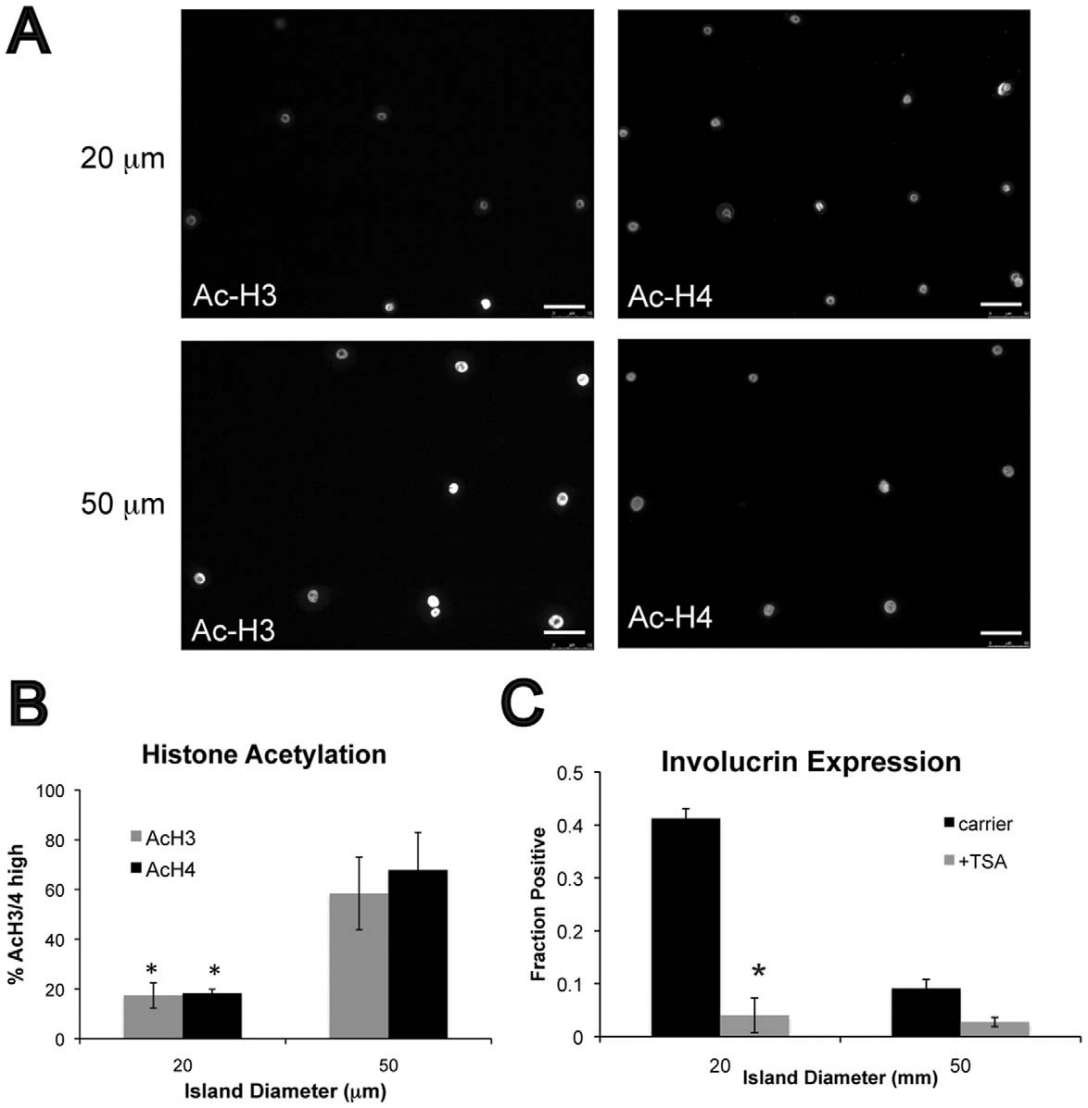
Role of global histone acetylation in shape-induced differentiation. (A) Representative immuno-fluorescence images of acetylated H3 (Ac-H3) and H4 (Ac-H4) in cells after 24 h on micro-patterned substrates containing 20 µm or 50 µm diameter islands. (B) Quantification of Ac-H3 and Ac-H4 on patterned substrates. Data are means ± SEM of 3 experiments. *P<0.05 compared to 50 µm islands. (C) Quantification of involucrin positive cells 24 h after plating on patterned substrates and treatment with carrier or 200 nM TSA. Data are means ± SEM of 3 experiments. *P<0.05 compared to carrier.

To directly determine the effects of restricted adhesion on histone acetylation we next examined global H3 and H4 acetylation by immunofluorescence labeling of cells on micro-patterned substrates. We observed a significant reduction in the number of keratinocytes with hyper-acetylated H3 and H4 on the 20 µm islands compared to the 50 µm islands (Fig. 6A, B). In addition, TSA treatment completely blocked shape-induced involucrin expression on 20 µm islands (Fig. 6C). We conclude that restricted adhesion leads to global histone de-acetylation and this chromatin modification is required for terminal differentiation.

**Figure 7:**
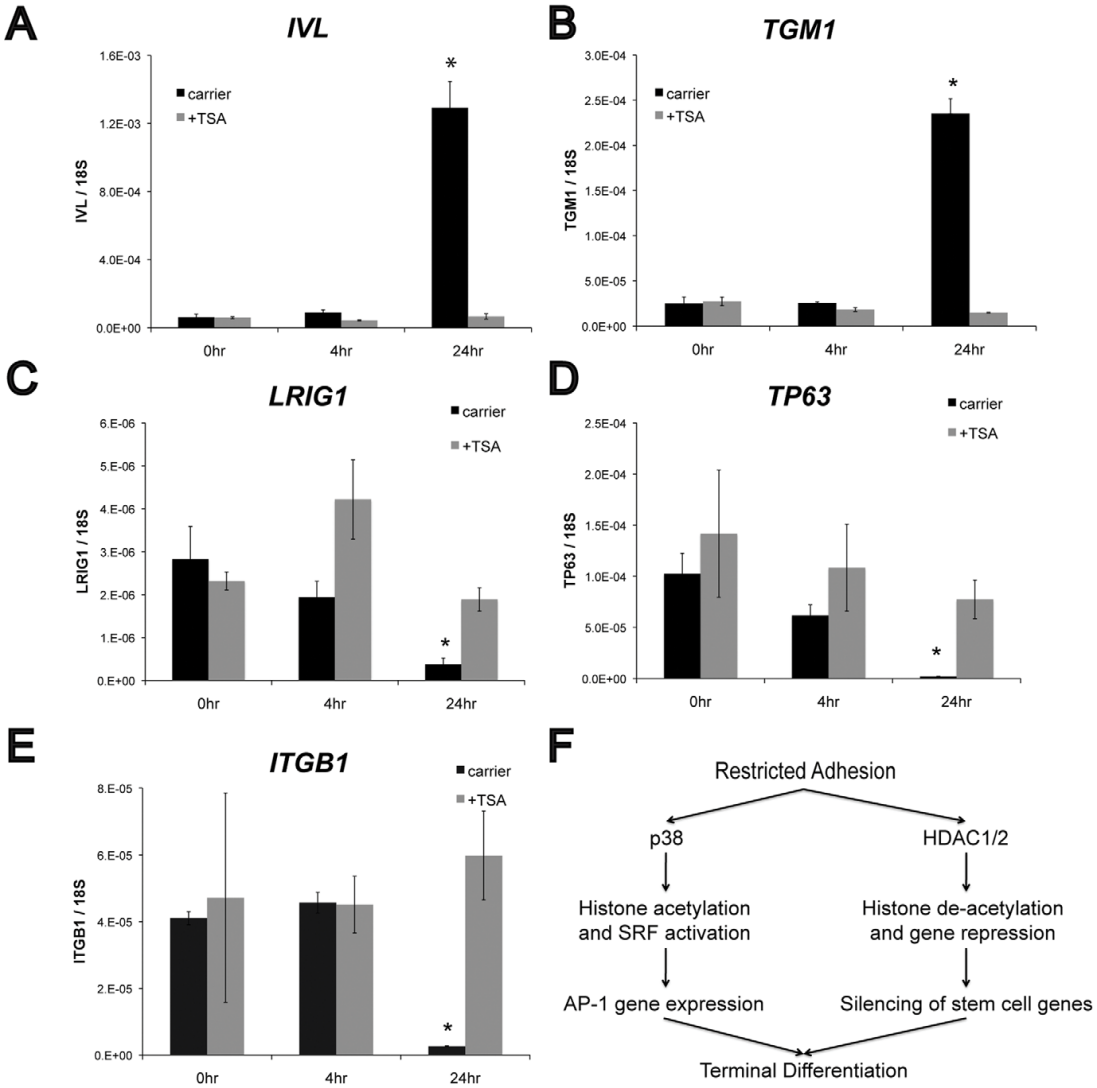
Effect of TSA on suspension-induced differentiation. Keratinocytes were cultured for 24 h in 1% methylcellulose with or without 200 nM TSA. Expression of (A) *IVL*, (B) *TGM1*, (C) *LRIG1*, (D) *TP63*, and *ITGB1* mRNA levels were measured by real-time RT-PCR at 0, 4, and 24 h. Data are means ± SEM of 3 experiments. *P<0.05 compared to carrier. (E) Proposed model for the role of histone acetylation during shape-induced terminal differentiation of human keratinocytes.

The inhibitory effects of TSA on involucrin expression were surprising given that it also stimulated *FOS* and *JUNB* gene expression, which are required for terminal differentiation. We hypothesized that up-stream regulatory genes were also activated by histone acetylation to suppress differentiation. We therefore examined the effect of TSA on *LRIG1*, *TP63,* and *ITGB1*. When keratinocytes were disaggregated and placed in suspension, mRNA levels of the differentiation markers involucrin (*IVL)* and transglutaminase I (*TGM1)* were significantly upregulated after 24 h (Fig. 7A, B). This response was completely blocked by TSA, consistent with the effects of TSA on cells on micro-patterned substrates. During suspension-induced differentiation *LRIG1*, *TP63*, and *ITGB1* mRNAs were down-regulated in untreated cells; however, TSA treatment maintained expression of these genes at nearly 0 h levels (Fig. 7C–E). These findings suggest that histone deacetylation in human keratinocytes is required to repress stem cell genes during terminal differentiation.

## Discussion

Our study demonstrates that keratinocyte terminal differentiation requires a coordinated program of histone acetylation and de-acetylation (Fig. 7F). Histone acetylation enhances SRF transcriptional activity and expression of AP-1 factors, which are required for differentiation, and this response is mediated by p38 MAPK. At the same time, we observe a global reduction in histone acetylation upon shape-induced differentiation. Inhibiting HDACs completely blocked involucrin expression while maintaining expression of known markers of epidermal stem cells. Furthermore, p38 inhibition did not affect *LRGI1*, *DLL1*, or *TP63* expression and reduced SRF transcriptional activity even in the presence of TSA. Histone acetylation therefore appears to play a dual role in gene silencing and activation during terminal differentiation and is regulated by at least two independent pathways.

Cellular stresses, such as ultra-violet light [27] or loss of adhesion [28], can stimulate p38 phosphorylation in human keratinocytes. Consistent with our findings, several reports describe a role for p38 in keratinocyte terminal differentiation [29], [30]. While the SB202190 compound only targets the α and β isoforms, some reports suggest that the δ isoform is primarily responsible for regulating involucrin expression [9]. It will be interesting to explore the specific role of each isoform in SRF signaling and histone acetylation in future studies.

The SRF target genes we examined displayed differential sensitivity to p38 inhibition during serum-stimulation and restricted adhesion. While serum stimulation of *JUNB* and *CTGF* was delayed by SB202190, *FOS* was unaffected and *EGR1* actually increased. Similarly, *JUNB* but not *FOS* was inhibited by SB202190 on the micro-patterned substrates. It is interesting to note that p38 inhibition had a greater effect on *JUNB* expression on the patterned substrates compared to serum stimulation. It has also been reported that p38 mediates *FOS* expression in response to UVB exposure, but not in response to serum stimulation [31]. These context dependent differences in gene regulation highlight how specific stimuli can activate AP-1 expression by different pathways and the complexity of their regulatory mechanisms.

In a recent study, p38 stimulated expression of the SRF target gene *CCN1* via the histone acetyl transferase activity of CREB binding protein (CBP) [26]. Genome wide analyses have also mapped CBP bindings sites to SRF and AP-1 genes [32]. Thus, CBP may connect p38 activity with histone acetylation of SRF target genes. Interestingly, the CBP homolog p300 is required for keratinocyte terminal differentiation and influences p21 expression by histone acetylation; however, this response is not affected by CBP knockdown [32]. While it is clear from our studies and others that CBP and p300 play important roles in histone acetylation and keratinocyte differentiation, further work is needed to elucidate their specific functions and regulatory mechanisms.

In the present study we employed micro-patterned substrates to manipulate keratinocyte shape and adhesion. While this system has the advantage of precisely controlling single cell behavior, it is by nature highly synthetic and in some cases not representative of the in vivo situation. For example, we observe a direct transition from stem cells to terminal differentiated keratinocytes while maintaining adhesion to a basal ECM. In contrast, cells in vivo lose contact to the basement membrane before turning on late terminal differentiation markers, such as involucrin. While it is important to keep these differences in mind when interpreting the results of our studies, this model system has allowed us to identified SRF as a key regulator of terminal differentiation [6]. This result has been corroborated by two different mouse models [8], [33] and demonstrates that our approach can provide new and physiologically relevant insights into keratinocyte terminal differentiation.

Histone acetylation regulates the differentiation and self-renewal of multiple types of stem cells. In embryonic stem cells, the nucleosome remodeling and de-acetylation (NuRD) complex, is required for maintaining pluripotency [34], and loss of HDAC1 causes spontaneous differentiation along cardiac and neuronal lineages [35]. In contrast, deletion of HDAC 1 and 2 in mice inhibits the differentiation of neural pre-cursers into differentiated neural lineages [36]. These findings indicate that the role of histone acetylation in differentiation depends on the specific cell type and stage of development. Interestingly, Myc-stimulated differentiation in the mouse epidermis is associated with histone acetylation [19], yet deletion of HDAC1 and 2 in vivo [18] or TSA treatment in our in vitro model blocks terminal differentiation. Together, these findings further suggest that histone acetylation has distinct effects on cell behavior at different stages of epidermal differentiation.

Although the functions of several chromatin remodeling complexes have been described in the skin [16], [18], we still know very little about the upstream signals governing their activity. Cell rounding has previously been shown to promote global histone de-acetylation in mammary epithelial cells [37] and tensile stretching can stimulate CBP acetyl-transferase activity in fibroblasts [26]. Our work is the first to implicate extrinsic physical cues in regulating chromatin modifications that are essential for stem cell differentiation. The mechano-transduction mechanisms leading to chromatin remodeling are likely to be important regulators of transcription during other physical processes, including wound healing, neuronal out-growth, and tumor invasion.

## Materials and Methods

### Generation of micro-patterned, polymer brush substrates

Patterned poly-oligo(ethylene glycol methacrylate) (POEGMA) brushes were generated as previously described [6], [7], [38]. Briefly, master silicon molds were created by photolithography and used to cast poly-dimethylsiloxane (PDMS) stamps. The micro-patterned stamps were inked with the thiol initiator, ω-mercaptoundecyl bromoisobutyrate, and brought into conformal contact with gold-coated coverslips to deposit the initiator as a self-assembled monolayer. Atom transfer radical polymerisation (ATRP) of oligo ethylene glycol methacrylate (MW 300) was carried out in a water/ethanol (4∶1) solution of OEGMA (1.6 M), Cu(II)Br_2_ (3.3 mM), bipyridine (82 mM), and Cu(I)Cl (33 mM). The reaction was performed at room temperature for 15 minutes, resulting in an estimated 20 nm thick brush. The patterned substrates were rinsed with water and ethanol, dried, and stored under N_2_. Immediately before cell seeding, patterned substrates were coated with 20 µg/ml of rat type I collagen (BD Biosciences) in phosphate-buffered saline (PBS) for 1 h at 37°C. Substrates were rinsed three times with 1 mM HCl and twice with PBS.

### Culture of primary human keratinocytes

Primary human keratinocytes were isolated from neonatal foreskin. All the cells used in this study were obtained prior to the Human Tissue Act 2006, and the patients were kept anonymous. Keratinocytes (KY strain passage 2–8) were cultivated on a feeder layer of J2 3T3 fibroblasts as previously described [39]. Following removal of the feeder layer, keratinocytes were trypsinized and re-seeded onto the micro-patterned substrates at a density of 25,000/cm^2^ in complete FAD (1 part Ham's F12, 3 parts DMEM, 10^−4^ M adenine, 10% FBS, 0.5 µg/ml hydrocortisone, 5 µg/ml insulin, 10^−10^ M cholera toxin, 10 ng/ml EGF) medium. Cells were allowed to adhere for 1 h and rinsed three times with fresh medium. For some experiments, inhibitor supplemented medium was added at the indicated dose immediately after rinsing. For suspension culture experiments, keratinocytes were resuspended in complete FAD supplemented with 1% methylcellulose and cultured for to 24 h before recovery by centrifugation.

### Antibodies and inhibitors

Rabbit anti-Ki67 was purchased from Abcam (Cambridge, UK). Mouse anti-involucrin (SY5), and mouse anti-transglutaminase I (BC1) were prepared by Cancer Research UK central services. Rabbit anti-AcH3, anti-AcH4, and anti-total H3 were purchased from Millipore,. Cytochalasin D, PD98059, PD169316, SB202190 and SP600125 were obtained from Calbiochem (La Jolla, CA), and Trichostatin A was from Sigma Aldrich (St. Louis, MO).

### Transfections and luciferase assays

Keratinocytes were cultured in KSFM (Gibco) on collagen-coated dishes for 24 h prior to transfection. Cells were transfected for 3 h using 1 µl Lipofectamine 2000 (Invitrogen) and 1 µg of DNA per 10^5^ cells. The FLAG-MAL and SRF reporter (p3DA.luc) constructs were kindly provided by Richard Treisman and have been described previously [10]. Thymidine kinase (TK) driven Renilla was used as an internal control and transfected 1∶1 with p3DA.luc. Following DNA transfection, cells were rinsed and cultured for 24 to 48 h before treating with FBS or inhibitors. Luciferase assays were carried out in 24-well plates (n = 4 wells). Cells were treated for 7 h with FBS and the indicated doses of inhibitors, then harvested and analyzed using the dual luciferase assay (Promega). SRF activity was reported relative to thymidine kinase levels, except in the TSA experiments. In this case, TSA significantly affected the control reporter and only raw SRF activity was reported.

### Immunofluorescence staining and quantification

Cultured cells were fixed in 4% PFA for 10 minutes and permeabilized with 0.2% Triton-X100 for 5 minutes at room temperature. Samples were blocked for 1 h in 10% FBS plus 0.25% gelatin, incubated with primary antibodies for 1 h at room temperature or overnight at 4°C, and incubated with Alexafluor (488 and 555)-conjugated secondary antibodies for 1 h at room temperature. TRITC-phalloidin and DAPI were included in the secondary antibody solution where indicated. Fixed and stained coverslips were mounted on glass slides with Mowiol reagent, and images were acquired with a Leica DMI4000 fluorescence microscope or Leica TCS SP5 confocal microscope. For scoring involucrin, transglutaminase 1, and AcH3/4 expression at least 6 fluorescence images were taken per condition (approximately 50 cells) per experiment and analyzed with Image J software. Cells with intensities above background levels (normal mouse serum) were scored as positive.

### Western blotting

Keratinocytes were lysed in ice cold RIPA buffer with protease inhibitor tablets (Roche). Insoluble material was removed by centrifugation, and the supernatent was diluted with a loading buffer (Invitrogen) containing 1% 2-Mercaptoethanol. Equal amounts of lysate were separated by SDS-PAGE on a 4–12% gradient gel (Invitrogen), and transferred to PVDF membranes. Membranes were blocked with 5% non-fat dry milk, and incubated overnight with primary antibodies for Ac-H3 (1∶1000) or total H3 (1∶1000). Secondary detection was performed with a HRP-conjugated anti-rabbit antibody (1∶5000) at room temperature for 1 h. Blots were treated with ECL reagent (GE Healthcare) for 1 min and developed on X-ray film.

### Chromatin immunoprecipitation

Chromatin immunoprecipitation was performed as described previously [6]. Keratinocytes (10^7^) were cultured overnight in KSFM with or without inhibitors and fixed with 1% PFA for 10 min. Chromatin was sonicated to an average length of 1 kb, incubated overnight with 10 µg of anti-FLAG, anti-SRF, or anti-AcH3 antibodies at 4°C, and precipitated with G-protein coupled Dynabeads (Invitrogen) for 4 h at 4°C. Cross-links were reversed by incubating at 65°C overnight, and DNA was isolated with a PCR purification kit (Qiagen). Quantitative RT-PCR was performed with Sybr Green (Sigma) and custom primers for regions around *JUNB, FOS, SATCEN11*. Data are reported as the fold enrichment compared to the anti-FLAG control.

### Real-time RT-PCR

Total RNA was isolated using a Purelink RNA kit (Invitrogen) and reverse transcribed to cDNA using a Superscript III kit (Invitrogen). PCR reactions were carried out with Taqman Gene Expression Assays for *FOS, JUNB, CTGF, EGR1, IVL, TGM1, LRIG1, DLL1, TP63,* and *ITGB1* (Applied Biosystems), and all data were normalized to *GAPDH* or 1*8S* expression.

### Statistical Analyses

All data were analyzed by one or two factor ANOVA and Tukey's test for posthoc analysis. Significance was determined by P<0.05.
